# 12.1 CELL-SUBTYPE SPECIFIC BIOENERGETIC DEFECTS IN SCHIZOPHRENIA

**DOI:** 10.1093/schbul/sby014.044

**Published:** 2018-04-01

**Authors:** Courtney Sullivan, Robert McCullumsmith

**Affiliations:** University of Cincinnati

## Abstract

**Background:**

Novel insights into the pathophysiology of schizophrenia are needed to move the field forward by providing the conceptual framework to facilitate development of new treatment strategies. It is well established that glutamatergic systems are disrupted in schizophrenia, which are intimately linked to metabolic function. While there are many promising new directions, accumulating evidence suggests that bioenergetic function is impaired in the brain in schizophrenia. There are multiple mechanisms in the brain to meet neuronal energy demands, including glycolysis, lactate uptake, and oxidative phosphorylation. In normal brain, neurons and astrocytes are coupled through the astrocyte-neuron lactate shuttle, where astrocytes metabolize glucose to lactate and pyruvate, primary energy substrates that are transported to neurons via monocarboxylate transporters (MCTs). Lactate generated by glycolysis in glial cells constitutively supports synaptic transmission even under conditions in which a sufficient supply of glucose and intracellular adenosine triphosphate (ATP) are present. Interestingly, working memory and other cognitive domains are dependent on the shuttling of lactate from astrocytes to neurons. This process highlights the bioenergetic coupling between astrocytes and neurons that develops as the brain matures, forming a critical biological process in the mature adult brain. We assessed elements of these systems in postmortem brain, testing the hypothesis that there are cell-subtype defects in bioenergetics function in the frontal cortex in schizophrenia.

**Methods:**

Well-validated assays were used to assess the activity of three glycolytic enzymes in postmortem dorsolateral prefrontal cortex (DLPFC) samples (n=16/group): lactate dehydrogenase (LDH), hexokinase (HXK), and phosphofructokinase (PFK). Each sample was assayed with and without a specific inhibitor (in duplicate) and normalized to protein loaded into the assay. We also probed for differences in protein expression using western blot analysis. Western blot analyses were run in duplicate using the following antibodies optimized for postmortem brain: MCT1, LDH, LDHA, LDHB, HXK1, glucose transporter 3 (GLUT3). We performed real time quantitative polymerase chain reaction (RT-qPCR) using TaqMan PCR assays (MCT1, MCT4, HXK1, HXK2, LDHA, LDHB, PFK1, GLUT1, and GLUT3) in duplicate on cDNA samples in 96-well optical plates on a Stratagene MX3000P (Stratagene, La Jolla, California). We also coupled laser capture microdissection (LCM) with RT-qPCR from superficial and deep layers of DLPFC using the Veritas Microdissection instrument and CapSure Macro LCM caps (Life Technologies, formerly Arcturus, Mountain View, CA, USA). Similar studies were performed in haloperidol-decanoate or vehicle (sesame oil) treated rats (intramuscular injection every 3 weeks for 9 months).

**Results:**

We found a 24% decrease in PFK1 mRNA expression in the dorsolateral prefrontal cortex in schizophrenia (p=0.039). We also found decreases in HXK (26%, p=0.002) and PFK (16%, p<0.001) activity in the dorsolateral prefrontal cortex. These changes were not present in haloperidol treated rats. At the cell-level, in pyramidal neurons we found an increase in MCT1 mRNA expression (22%, p= 0.038), and decreases in HXK1 (19%, p= 0.023), PFK1 (22%, p=0.003), GLUT1 (20%, p=0.008), and GLUT3 (20%, p=0.023) mRNA expression. We found increases in MCT1 (17%, p<0.05) and GLUT3 (20%, p<0.05), but not HXK1, PFK1, or GLUT1, mRNA expression in enriched pyramidal neuron samples of antipsychotic treated rats.

**Discussion:**

As the brain develops, bioenergetic organization and the formation of synapses occur simultaneously, creating a fundamentally interdependent system. There is accumulating evidence of implicating a number of abnormalities associated with glucose metabolism, the lactate shuttle, and bioenergetic coupling in schizophrenia, suggesting energy storage and usage deficits in the brain. Bioenergetic deficits and genetic risk for synaptic dysfunction in schizophrenia could contribute to the pathophysiology of this illness. In normal brain, glucose enters cells through GLUTs and is processed by glycolytic enzymes resulting in bioenergetic substrates such as pyruvate. Pyruvate can then be converted to lactate and transported between cells or intracellularly by MCTs to be oxidized in the TCA cycle when neuronal energy demand is high. Our findings of decreased glycolytic enzyme and lactate transporter mRNA expression suggests a decrease in the capacity of pyramidal neurons to generate bioenergetic substrates from glucose via glycolytic pathways. Additionally, if neurons were unable to take up adequate amounts of glucose for glycolysis, the intracellular pool of available pyruvate/lactate for transport into mitochondria may be diminished, ultimately impacting energy supply. It is also possible that there is attenuated glycolysis in pyramidal neurons, with a shift towards pathways that boost protection from oxidative stress (pentose phosphate pathway). Other studies also report region and cell-subtype specific changes in the expression of genes encoding proteins involved in metabolism in this illness. Importantly, the above changes were not attributable to antipsychotic treatment. Both synaptic function and meeting of energetic demands are essential for cognition, and failure of either could contribute to the cognitive symptoms seen in schizophrenia. Augmenting affected systems such as glucose utilization pathways could offer a novel approach to restoring cognitive function in schizophrenia. This could include targeting pro-metabolic substrates pharmacologically.

